# Fashionable Freaks

**Published:** 1881-08

**Authors:** 


### FASHIONABLE FREAKS.

WHEN the French nation reached its height of folly and wickedness, just before the revolution broke out and flooded the land with misery and bloodshed, all who desired to be considered connected with the aristocracy carried about with them at least one *pantin.* These were small wooden dolls which, by pulling a string, suddenly jerked out arms and legs, exactly like those which may be seen adorning the hats of "swells" on a Derby day. The rage for them was immense. Nobles, gentlemen, and even grave ecclesiastics were to be seen carrying them about and playing with them. A somewhat similar rage for comfits existed in the reign of Henry III. of France. When the body of the Duc de Guise was found after the battle of Blois he had a comfit-box in his hand. In 1585 the ladies carried hand mirrors attached to their chatelains, and like Narcissus were perpetually admiring their own charms. This excited the deepest indignation of Jean des Caures, a stern old moralist of the time, and he emphatically menaced them with the extremest penalties of the other world. Who would have believed that so late as 1751 the dress of a dandy should have consisted of a black velvet coat, a green and silver waistcoat, yellow velvet breeches and blue stockings! A satirical writer of about the same period gives a biting sketch of one of his contemporaries: "A coat of light green, with sleeves too small for the arms, and buttons too big for the sleeves; a pair of Manchester fine-stuff breeches, without any money in the pockets; clouded silk stockings, but no legs; a club of hair behind larger than the head that carries it; a hat of the size of a sixpence on a block not worth a farthing." No doubt the same gentleman could paint a picture of the dress of our own time which would appear as ridiculous to the gentleman with the green coat as his own does to us.—*Chambers' Journal.*

### A LAND WITHOUT BIRDS.

A French novelist somewhere says of the Englishman: "Let us go out and kill something!" This is his idea of the Englishman's practice. But he forgets his own countrymen, remarks an exchange. We have still kept our birds, though many have been destroyed by cold and hunger during these latter winters, and many more by shooting and battues. Still, our birds are the glory of the land—Gloria in excelsis! But in France the fields are mute. There is no music from the skies. The larks have been netted and eaten. The birds of gay plumage have been shot and their wings put in ladies' bonnets. All over the country sparrows, finches, robins and nightingales, have disappeared. All are killed and eaten. But now comes the punishment. The trees are eaten bare; the vines are destroyed by phylloxeræ; the leaves of the shrubs are destroyed by caterpillars. They are seen hanging in bunches from the trees. The birds have been killed that destroyed the grubs and phylloxeræ. Hence destruction is spreading over France. The crops are eaten up to the roots, and the vines are in some districts entirely fruitless. Thus inhumanity, like curses, comes home to roost. Waterton has calculated that a single pair of sparrows destroy as many grubs in one day as would have eaten up half an acre in a week. And the London *Times* says: For the matter of birds, France is a dark and silent land. The eye searches in vain, the ear listens in vain, for nature there sits lamenting her children that are not. Whatever may be said of Republican institutions, they can claim no partnership with nature, who clings to her old friends, feudalism and aristocracy. If there were reported anywhere in France as great a number of birds of gay plumage and trilling song as may be seen and heard almost anywhere a few miles from the metropolis, populations would turn out in fancy costumes, carrying guns and large bags, followed by nondescript dogs, and ready to watch whole days for the chance of a victim within easy range.

In Italy birds are used for the amusement of children. A string is tied to the bird's leg. When the bird tries to fly, it is pulled down by the string. When his powers of flight are exhausted, it is generally plucked alive and dismembered. The children do not understand that a beast or a bird can be a fellow-creature. When expostulated with, they answer, "Nona Cristiana"—It is not a Christian.

